# Development of an Ion Chromatography Method for Analysis of Organic Anions (Fumarate, Oxalate, Succinate, and Tartrate) in Single Chromatographic Conditions

**DOI:** 10.3797/scipharm.1503-15

**Published:** 2015-05-29

**Authors:** Yarbagi Kaviraj, B. Srikanth, J. Moses Babu, B. Venkateswara Rao, S. Paul Douglas

**Affiliations:** 1 Analytical Research, Custom Pharmaceutical Services, Dr. Reddy’s Laboratories Ltd., Bollaram road, Miyapur, Hyderabad-500049 (AP), India; 2 Department of Engineering Chemistry, Andhra University, Visakhapatnam-530003, India

**Keywords:** Ion chromatography, Anions, Validation, Fumarate, Oxalate, Succinate, Tartrate

## Abstract

A single organic counterion analysis method was developed by using an ion chromatography separation technique and conductivity detector. This allows the rapid characterization of an API to support clinical studies and to fulfil the regulatory requirements for the quantitation of fumarate, oxalate, succinate, and tartrate counterions in active pharmaceutical ingredients (quetiapine fumarate, escitalopram oxalate, sumatriptan succinate, and tolterodine tartrate). The method was developed by using the Metrohm Metrosep A Supp 1 (250 × 4.0 mm, 5.0 µm particle size) column with a mobile phase containing an isocratic mixture of solution A: 7.5 mM sodium carbonate and 2.0 mM sodium bicarbonate in Milli-Q water and solution B: acetonitrile. The flow rate was set at 1.0 mL/min and the run time was 25 minutes. The developed method was validated as per ICH guidelines, and the method parameters were chosen to ensure the spontaneous quantitation of all four anions. The method was validated for all four anions to demonstrate the applicability of this method to common anions present in various APIs.

## Introduction

A salt is a “chemical compound comprising an assembly of cations and anions.” Thus, a pharmaceutical salt comprises an active pharmaceutical ingredient (API) that is molecular and either cationic or anionic and has a counterion that might be molecular or monatomic [1]. Analysis of anions in APIs is carried out for two reasons. The first is to demonstrate the quantification of an appropriate amount of anionic counterion in the salt, which is an important step in the characterization of an API [2, 3].

The second reason is to assess the amounts of anionic synthetic impurities and degradation product, which is important in understanding degradation pathways, drug stability, and in establishing the re-test period to determine shelf-life [4–7].

Some of the common quantification methodologies to analyze anions in APIs include potentiometric titrations [3], ion-selective electrodes [8–10], complexometric methods, chromatographic methods with indirect UV detection [11, 12], capillary electrophoresis methods with indirect UV detection [3, 13, 14], chromatographic methods with light scattering detection [3], and chromatographic methods with suppressed [3, 4, 6] and non-suppressed conductivity detection [2]. The commercial availability of integrated instrumentation, availability of appropriate chromatographic conditions and columns, and high sensitivity led to the selection of ion exchange chromatography with suppressed conductivity detection, also known as ion chromatography (IC).

It is important to determine the concentration of the counterion in the drug substance, because the determination of the counterion is essential to establish the stoichiometry, the correct molecular mass of the drug, and the completeness of salt formation. Furthermore, counterion determination is also important in drug authenticity studies. This work describes the development of chromatographic parameters and sample preparation procedures for a single method for the quantification of fumarate, oxalate, succinate, and tartrate by IC. This work also describes the analytical method validation of the IC method for use in regulated environments [15–18].

## Experimental

### Chemicals & Reagents

**Fig. 1:**
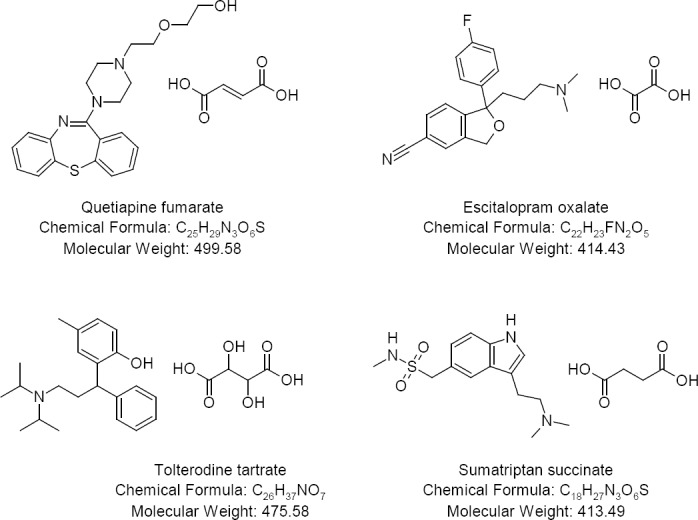
APIs used in this study

Analytical grade sodium carbonate and sodium bicarbonate were purchased from S.D. Fine Chemicals, Mumbai, India. Analytical reagent grade sulphuric acid and acetonitrile were purchased from Merck, Mumbai, India. High-purity water was collected from a Millipore Milli-Q water purification system (Millipore, Milford, MA, USA). Analytical grade fumaric acid, oxalic acid, succinic acid, and tartaric acid were purchased from Qualigens Fine Chemicals, Mumbai, India. Quetiapine fumarate, escitalopram oxalate, sumatriptan succinate, and tolterodine tartrate were the APIs for research (Fig. 1), which were obtained from Dr. Reddy’s Laboratories Ltd, Hyderabad, India.

### Equipment

The ion chromatography system was purchased from Metrohm, Herisau, Switzerland and used throughout this study, which was equipped with the 818 IC pump, 833 liquid handling unit, sampling injector with a 20 µL loop, 820 IC separation centre equipped with a cation suppressor, and a conductivity detector. Quantitation was performed from the output signal, monitored, and processed using the IC Net 2.3 SR4 version software on a Compaq computer (Digital Equipment Co). Dilutions were accomplished with Hamilton Precision Pipettes (Bondaiz, Switzerland).

### Chromatographic Conditions

The chromatographic column used was a Metrosep A Supp 1 column (250 × 4.0 mm, 5.0 µm particle size) having a stationary phase of polyvinyl alcohol with quaternary ammonium groups [16], that was safeguarded with Metrosep A Supp 4/5 guard column. The mobile phase used was a mixture of 7.5 mM sodium carbonate and 2.0 mM sodium bicarbonate prepared in HPLC grade water, and then it was mixed with acetonitrile in the ratio of (90:10). The flow rate of the mobile phase was set at 1.0 mL/min. The injection volume was 20 µL. The diluent used was Milli-Q water: acetonitrile (80:20).

The anion exchange chromatographic system was equipped by a cation exchange resin suppressor for chemical suppression. Chemical suppression reduces the background conductivity and replaces the counterions in the sample, i.e. all cations from the mobile phase are replaced by H^+^. By this suppression reaction, an eluent with high conductivity is transferred to water and carbon dioxide which has low conductivity. The suppressor is regenerated after each run using a suppressor regenerator followed with suppressor rinsing with HPLC grade water. The suppressor regenerator used is 50 mM sulphuric acid prepared in Milli-Q water. The detector interface was set with a detector range of 100 μS/cm and a detector full scale of 20 μS/cm. The run time for each run was 25 min.

### Preparation of Solutions

The standard solutions were prepared by dissolving the fumaric acid, oxalic acid, succinic acid, and tartaric acid in Milli-Q water at a 20 µg/ mL concentration. The test solutions for quetiapine fumarate, escitalopram oxalate, sumatriptan succinate, and tolterodine tartrate were prepared by dissolving them in Milli-Q water at 134 µg/ml, 100 µg/mL, 66 µg/mL, and 66 µg/mL concentrations, respectively. The standard solutions prepared at 20 µg/mL concentration corresponded to the theoretical contents of fumaric acid (15.13% w/w) in quetiapine fumarate, oxalic acid (21.24% w/w) in escitalopram oxalate, succinic acid (18.48% w/w) in sumatriptan succinate, and tartaric acid (31.27% w/w) in tolterodine tartrate.

### Method Development

Various trials were performed for the method development of organic anion content in quetiapine fumarate, escitalopram oxalate, sumatriptan succinate, and tolterodine tartrate. The trials were done to separate the peaks of interest from all other peaks of the test solution. Finally, the conditions were achieved as mentioned in the section “Chromatographic Conditions.”

### Method Validation

During method optimization, all chromatographic parameters were found to prove specificity, precision, linearity, accuracy, robustness, solution and mobile phase stability of fumarate, oxalate, succinate, and tartrate anions.

#### Specificity

Specificity is the ability to unequivocally assess the analyte in the presence of its potential impurities, which may be expected to be present like impurities, degradants, matrix, etc. The specificity of the developed ion-exchange chromatographic method was established in the presence of 11 anions and four active pharmaceutical ingredients (API), namely trifluoroacetic acid (TFA), chloride, nitrate, bromide, phosphate, sulphite, succinate, tartrate, sulphate, oxalate, fumarate and the APIs quetiapine, escitalopram, sumatriptan, and tolterodine.

Drugs were not subjected to forced degradation, as the impurities generated were organic moieties, which do not have any response in the ion-exchange chromatographic method.

#### Precision

The precision of an analytical procedure expresses the closeness of agreement between a series of measurements from multiple samplings of the homogenous sample under the prescribed conditions.

The precision for quetiapine fumarate, escitalopram oxalate, sumatriptan succinate, and tolterodine tartrate were checked at the 134 µg/mL, 100 µg/mL, 66 µg/mL, and 66 µg/mL anions, respectively, corresponding to the theoretical content anions, i.e. 15.3%, 21.2%, 18.5%, and 31.3% of fumaric acid, oxalic acid, succinic acid, and tartaric acid in quetiapine fumarate, escitalopram oxalate, sumatriptan succinate, and tolterodine tartrate, respectively. Method precision was performed on six different preparations of the test samples. The percentage relative standard deviation of the content of all four anions in the six preparations was calculated.

The intermediate precision of the method was also evaluated by a different analyst, different instrument, and on a different day.

#### Linearity

The linearity of an analytical test procedure is its ability to obtain test results within the given range which is directly proportional to the concentration of the analyte in the sample. The linearity of the method was checked at seven concentration levels: from 25 µg/mL to 200 µg/mL of fumaric acid, oxalic acid, succinic acid, and tartaric acid. The calibration curve was drawn by plotting the peak areas of all four acids against the corresponding concentrations. The correlation coefficients of the regression lines of the calibration curves were also calculated.

#### Accuracy

The accuracy of an analytical procedure expresses the closeness of agreement between the value which is accepted either as a conventional true value or an accepted reference value, and the expected value found. Standard addition and recovery experiments were conducted to determine the accuracy of the quantitation of fumaric acid, oxalic acid, succinic acid, and tartaric acid in quetiapine fumarate, escitalopram oxalate, sumatriptan succinate, and tolterodine tartrate samples. The study was carried out by weighing drug substances to attain 50%, 100%, and 150%. Theoretical concentrations of the anions in their respective prepared drug substances were injected in triplicate at each level. The % recoveries of all four acids were calculated from the slope and y-intercept of the calibration curve obtained.

#### Solution Stability and Mobile Phase Stability

The solution stability was carried out by keeping both test solutions and reference solutions in tightly capped volumetric flasks at room temperature for 72 h. The sample solutions were analysed at initial, 24 h, 48 h, and 72 h. The stability of the mobile phase was also carried out for 72 h by analyzing the freshly prepared reference solutions at initial, 24 h, 48 h, and 72 h. The mobile phase was kept constant during the study.

#### Robustness

The robustness of an analytical procedure is a measure of its capacity to remain unaffected by small, but deliberate variations in method parameters and provides an indication of its reliability during normal usage, and the flow rate of the mobile phase was 1.0 mL/min in the method. To study the effect of flow rate on system precision, it was changed by 0.1 units to 0.9 mL/min and 1.1 mL/min, while mobile phase components were held constant and the effect of flow rate was studied. The acetonitrile in the mobile phase composition was 10% in the method. To study the effect of % acetonitrile on the system precision, it was changed by 2% to 8% and 12%, while other components were held constant and the effect of the change in % acetonitrile was studied. The concentration of sodium carbonate and sodium bicarbonate was 7.5 mM and 2.0 mM in the method. To study the effect of concentration of sodium carbonate and sodium bicarbonate, these were changed by 0.75 units to 6.75 mM and 8.25 mM for sodium carbonate, and by 0.2 units to 1.8 mM and 2.2 mM for sodium bicarbonate, while the other components were held constant and the effect of these changes were studied.

Robustness was not studied for column temperature as the method employs the column equilibration at room temperature in an analytical laboratory.

## Results and Discussion

A simple, precise, linear, and accurate analytical procedure was developed with ion exchange, high-performance liquid chromatography with conductivity detection which enables the determination and simultaneous quantitation of fumarate, oxalate, succinate, and tartrate in quetiapine fumarate, escitalopram oxalate, sumatriptan succinate, and tolterodine tartrate with simple, standard, and robust chromatographic conditions and sample a preparation procedure at optimum cost.

**Fig. 2:**
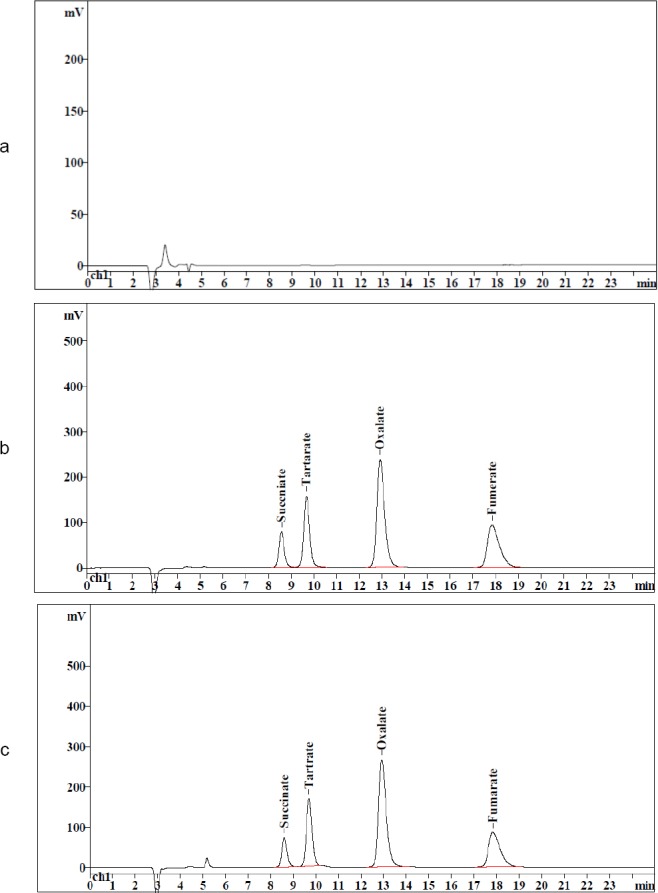
(a) Typical blank chromatogram, (b) standard anion chromatogram, (c) sample chromatogram showing the counterions succinate, tartrate, oxalate, and fumarate. The *y*-axis is the response in mV, and *x*-axis is the retention time in minutes. Mobile phase: 7.5 mM sodium carbonate and 2.0 mM sodium bicarbonate: acetonitrile (90:10), column: Metrosep A Supp 1 column (250 × 4.0 mm, 5.0 µm particle size), detector: conductivity

Satisfactory chromatographic peak shapes and consistent retention times were achieved with the accurately and scientifically selected mobile phase. The typical blank, standard solution, and test solution chromatograms are represented in Fig. 2.

### Specificity

**Fig. 3:**
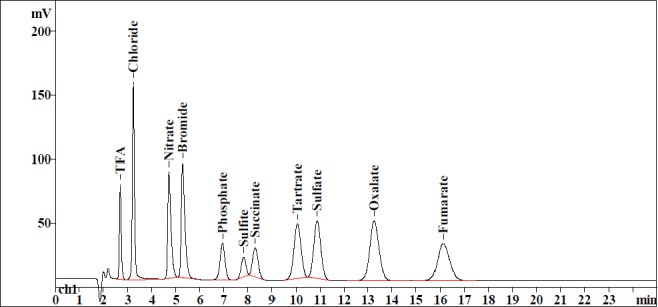
Specifity of succinate, tartrate, oxalate, fumarate in the presence of TFA, chloride, nitrate, bromide, phosphate, sulfite, and sulfate

There was excellent selectivity and specificity observed for the 11 anions. All four of the selected counteranions are well-resolved from each other and there was no interference either from the blank, other anions, or from their respective drug substances. The freebases of the drug substances, namely the quetiapine base, escitalopram base, sumatriptan base, and tolterodine base, being non-ionic, did not have any response in ion chromatography conductivity detection. The retention time details of the 11 anions are tabulated in Tab. 1 and chromatograms are shown in Fig. 3.

**Tab. 1:**
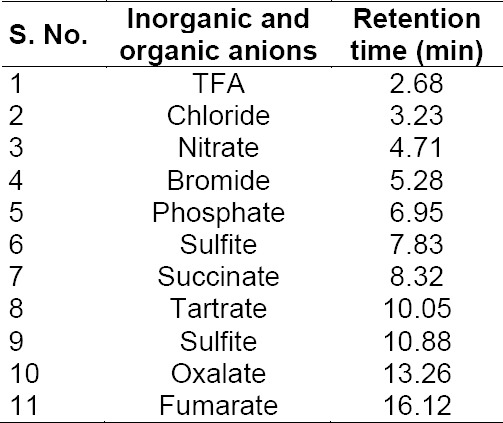
Inorganic and organic anions and their retention times

### Precision

The method precision of the analytical method for all four anions was checked at the specification level in their respective drug substances in six determinations of the test solution. The method precision experiments exhibited excellent results (% RSD) with very low % RSD.

The intermediate precision was also performed by a different analyst using a different instrument on different days. The results showed that the % RSD was less than 5.0%.

**Tab. 2:**
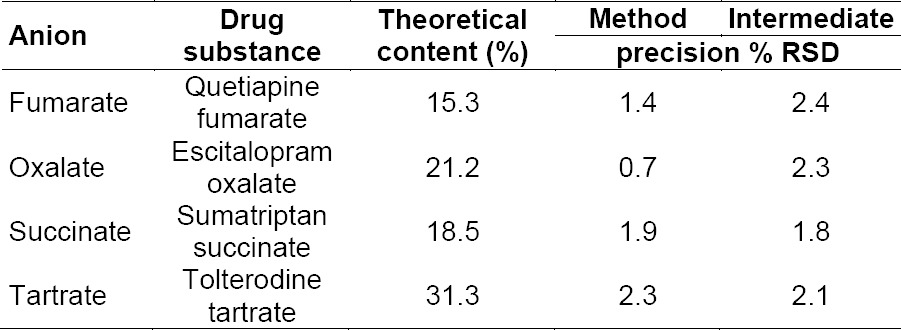
Precision results of anions

### Linearity

The linearity of the method for all four anions was checked at seven concentration levels: from 25 µg/mL to 200 µg/mL they exhibited results within the acceptance criteria. The linearity results are computed in Tab. 3.

**Tab. 3:**
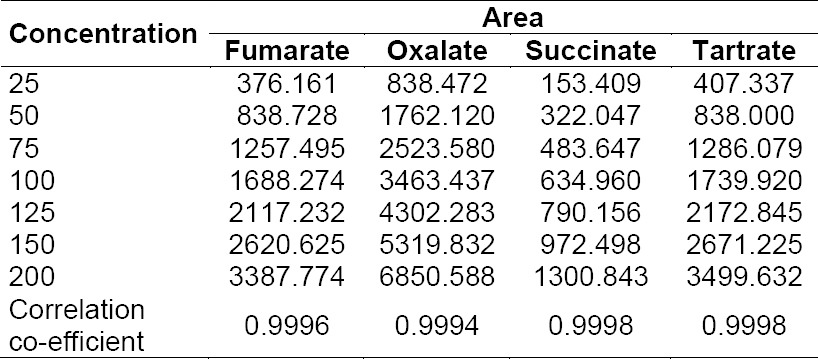
Linearity results

### Accuracy

The accuracy of the method for all four anions was checked by recovery experiments in the range of 50% to 150% of the specification level for each anions. Results were found to be close to the true value.

**Tab. 4:**
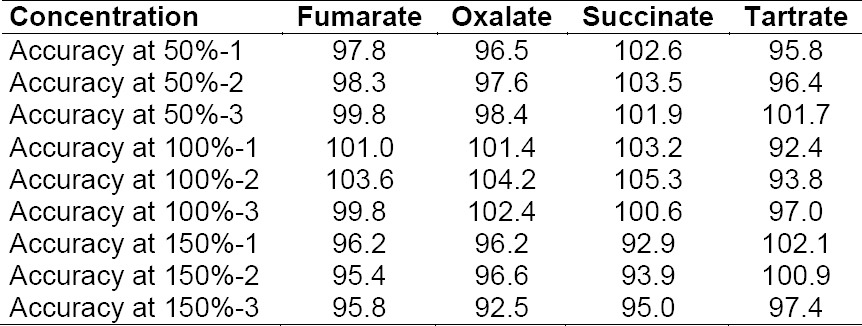
Evaluation of accuracy results (in %)

### Robustness

In all of the deliberately varied chromatographic conditions (flow rate, composition of acetonitrile, sodium carbonate, and sodium bicarbonate concentration), all analytes were adequately resolved and the elution order remained unchanged. The % relative standard deviation of the standard was less than 2.5; the % variation in the content was less than 0.41. The resolution between succinate, tartrate, oxalate, and fumarate was greater than 1.5. A very minor variation in the % RSD and % variation in the content was observed in all the robustness conditions. The results are shown in Tab. 5.

**Tab. 5:**
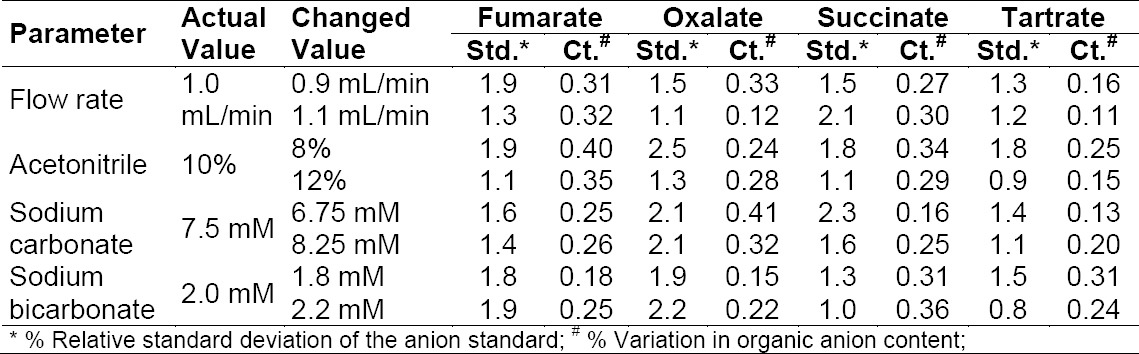
Results of the robustness parameter

### Solution Stability and Mobile Phase Stability

The variability in the estimation of all four counterions was within 10% during the solution stability and mobile phase stability tests. The results from the solution stability and mobile phase stability experiments confirmed that the mobile phase was stable up to 48 hr and the sample solution and standard solutions were stable up to 48 hr.

## Conclusion

The chromatographic and sample preparation conditions were developed and validated for organic counterions present in the active pharmaceutical ingredients. The method can perform the regulated analysis on minimal amounts (10–25 mg) of material. The method was validated for anions like fumarate, oxalate, succinate, and tartrate with appropriate accuracy, precision, linearity, and robustness. This method can be used for other pharmaceutical ingredients with minimal changes in method parameters: the IC method was shown to give results comparable to the reference methods while using considerably less material. Analysis of sulfonic acid counterions and sulfonate alkyl esters’ potential genotoxic impurities by ion chromatography will be the subject of future work.
